# Predictive value of 3D imaging to guide implant selection in immediate breast reconstruction

**DOI:** 10.1016/j.jpra.2021.10.007

**Published:** 2021-10-29

**Authors:** Monica Yu, Mary-Helen Mahoney, Gordon Soon, Brian Pinchuk, Ron Somogyi

**Affiliations:** 1 University of Toronto, Division of Plastic and Reconstructive Surgery; 2 North York General Hospital, Toronto, Ontario, Canada; 3 University of Toronto, Department of Pediatrics; 4 University of Toronto, Division of General Surgery

**Keywords:** Breast Neoplasms, Mastectomy, Breast Implants, Regression Analysis, Imaging, Three-Dimensional

## Abstract

**Background:**

Pre-operative estimation of breast mound volume for immediate breast reconstruction is necessary for operative planning, especially in direct-to-implant reconstruction. Our purpose was to investigate the relationship between pre-operative predictions of breast mound weight from 3D imaging and actual mastectomy weight and implant size.

**Methods:**

A retrospective chart review of all patients who had previously undergone nipple-sparing mastectomy (NSM) by a single surgeon was performed. Pre-operative 3D images were reviewed and calculations of breast mound weight were performed by three independent reviewers. Intra-operative mastectomy weight and final implant weight were collected from patient charts. A regression analysis between calculated and actual values was performed.

**Results:**

There were 59 reconstructed breasts included. Pre-operative 3D imaging-guided breast weight calculations were similar across reviewers (R=0.96). Pre-operative calculations of breast weight were 49.4g (SD=134.0) smaller than actual mastectomy specimens. Mastectomy specimens were 41.0g (SD=130.2) smaller than final implant sizes. Thereby, the relationship was as follows: Pre-operative calculated breast weight < actual Mastectomy weight < implant weight. Mastectomy weight and final implant size had linear relationships with pre-operative calculations of breast weight. Formulas for predicting mastectomy weight [mastectomy weight = 63.2 + 0.95 (pre-operative calculated weight)] and implant size [Implant weight = 209.7+ 0.56 (pre-operative calculated weight)] from pre-operative calculations of breast weight were generated.

**Conclusions:**

Three-dimensional scanning technologies may be a useful tool to predict implant sizes for direct-to-implant breast reconstruction. Final implant size was heavier than intra-operative mastectomy weight and pre-operative calculated breast mound weight.

## BACKGROUND

In the era of immediate breast reconstruction, oncologic surgeons and reconstructive surgeons are constantly looking for ways to better communicate expected outcomes. In addition, reconstructive surgeons learning or optimizing techniques for both implant and autologous immediate implant reconstruction often struggle with predicting the size necessary to achieve a reconstruction similar to the patients’ pre-surgical breast. In the case of direct-to-implant-based reconstruction, novice and expert surgeons alike must order a wide range of implants and sizers to aid in intra-operative implant selection that is ultimately based on the mastectomy weight.

Traditional mastectomy borders, however, may extend beyond what we consider to be the breast mound itself.^1^ These anatomic breast borders, as described by the American Society of Breast Surgeons, are: the sternal border medially, the clavicle superiorly, the latissimus laterally, and the rectus sheath/inframammary fold inferiorly.^1^ In many cases in our clinical experience, this results in a mastectomy weight that is greater than the predicted breast mound size. Significant discordance between the mastectomy weight and the predicted breast size adds difficulty to the reconstructive process intra-operatively as available implants may be inadequate and surgical mitigations, such as reinforcing borders may become necessary to prevent implant displacement. The relationship between the mastectomy weight (the “oncologic breast”) and the breast mound itself (the “aesthetic breast”) is not well understood. A better understanding of these differences may lead to better pre-operative prediction of volume replacement for breast reconstruction.

Three-dimensional modelling is used regularly in other domains of reconstructive surgery, such as craniofacial surgery.^2^ Pre-operative estimation of breast mound size can be achieved with multiple techniques, such as volume displacement or 3D imaging.^3^, ^4^, ^5^ Yip et al. demonstrated strong correlation between pre-operative breast volumes and mastectomy volumes by water displacement.^6^ Review of the published literature identified numerous studies validating 3D imaging technologies for breast volume assessments.^7^, ^8^, ^9^ However, we were only able to isolate one study using 3D imaging technologies to predict mastectomy and final breast implant volumes. This study by Utsunomiya et al. in 2017 was performed in a Japanese population undergoing two-stage breast reconstruction.^10^ Breast volumes were measured using the Microsoft Kinect 3D scanning technology and found that pre-operative breast volumes were very similar to mastectomy and implant volumes. Clinically, this finding diverges from our daily observations, whereby mastectomy weights (and thus, implant sizes) are larger than pre-operative breast size assessments. Notable differences between this previous study and our proposed study would include our diverse ethnic population, wider range of pre-operative breast sizes, Vectra 3D imaging technology, and differences in surgical education regarding mastectomy borders.

Our primary objective was to understand the difference in size between the oncologic breast, which is removed during mastectomy, and the aesthetic breast mound measured pre-operatively, which is to be reconstructed. Based on our clinical experiences, our hypothesis was that the oncologic breast weight is higher than the pre-operative breast mound size as determined by 3D imaging. If this difference is in a constant proportion, it may allow us to more accurately predict the implant size required for a given pre-operative breast size measurement.

## METHODS

### Study Design

The study design was a retrospective chart review of all patients who underwent nipple-sparing mastectomy (NSM) and implant-based immediate breast reconstruction from August 2014 to August 2018 in a single center by the primary investigator (RS). This study received institutional research ethics board approval (approval number: 18-0026). Office clinical records during this timeframe were reviewed to determine inclusion into the study. All patients who met inclusion and exclusion criteria stated below were included in this study. Inclusion criteria included any patient from ages ranging from 18 to 70, all ethnic backgrounds, diagnoses included anything for which a NSM is appropriate, including breast cancer, breast cancer gene positivity, or other high-risk status, and underwent direct-to-implant reconstruction. Exclusion criteria include patients who did not have 3D imaging pre-operatively, underwent non-NSM, and/or did not undergo breast reconstruction. Each independent breast was considered a separate subject for purposes of analysis.

Three-dimensional volume analysis was conducted using Vectra XT 3D Face and Body Imaging System (Canfield Scientific, Parsippany, NJ).

### Data Collection

**Figure 1:**
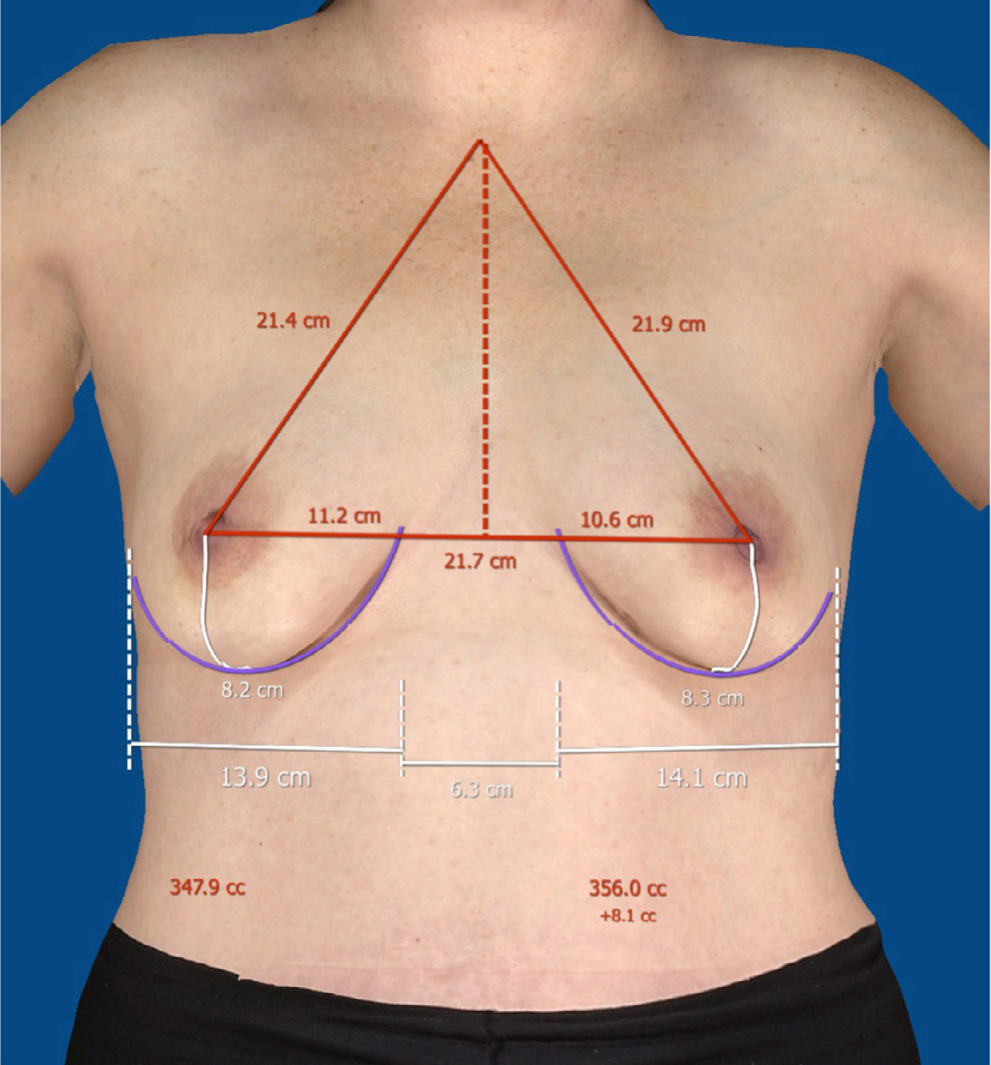
Calculation of pre-operative breast weight using Vectra XT 3D Face and Body Imaging System (Canfield Scientific, Parsippany, NJ).

Office charts were reviewed for patient history and physical examination details. Information abstracted included age, diagnosis, previous treatments, including previous surgery and radiation, degree of breast ptosis, incision type/location, implant volume, surgeon name, and surgery date(s). Hospital charts were accessed to obtain mastectomy weights and implant sizes. Three members of the research team independently selected breast landmarks on the 3D images to allow the Vectra software to calculate breast weight (see Figure 1). The mean value of the three calculated weights was determined prior to comparison with intra-operative mastectomy weights and final implant weights. The mean value of the pre-operative breast weight as determined by 3D imaging was then referred to as *calculated breast weight*.

All abstracted data were organized in an encrypted data collection sheet. Safeguards to protect patient privacy were maintained and all data were de-identified and stored in encrypted files. Data were aggregated for publication.

### Statistical Analysis

A sample size calculation was not performed. Given the retrospective nature of the study, all cases who met inclusion and exclusion criteria were reviewed. Data were analyzed using standard descriptive statistics. Pre-operative breast mound weights between independent reviewers were compared by calculating Pearson's correlation coefficient. A regression analysis for pre-operative breast mound weight, mastectomy weight, and implant weight was performed. Residuals of the regression model were calculated and analyzed. No outliers were removed from the data.

## RESULTS

**Figure 2:**
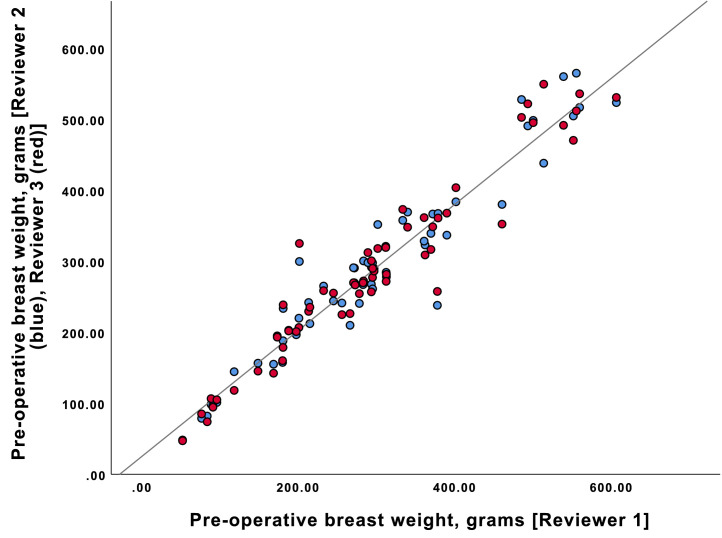
Correlation between independent reviewers for pre-operative breast weight

There were 59 breasts included in the study. The mean patient age was 49.8 years (SD=10.2) with a mix of cancer and prophylactic mastectomies. Breast ptosis varied between Regnault's grade 0 and 2 (see Table 1).^11^ Pre-operative breast weight as determined by 3D imaging was similar between the three independent reviewers – Pearson's correlation coefficient was statistically significant (*r* > 0.96, *n* = 59, *p* < 0.001; see Figure 2).

**Table 1:** Population demographics

	All (n= 59)
Age, in years	49.78 ± 10.16
Etiology (%)	
Cancer	24 (40.7)
Prophylactic	35 (59.3)
Ptosis, Regnault's grade (%)	
0	35 (59.3)
1	19 (32.2)
2	4 (6.8)

The mean calculated breast weight was 291.2g (SD = 127.7), the mean mastectomy weight was 332.7g (SD=178.0), and the mean implant weight was 373.8g (SD=106.3). Thus, the calculated breast weight was less than the actual mastectomy weight which was less than the implant weight. Comparing the mean differences showed us that the relationship between calculated breast weight and mastectomy weight was 49.4g (SD=134.0) and the difference between mastectomy weight and implant weight was 41.0g (SD=130.2; see Table 2). Thus, the relationship was as follows:

Calculatedbreastweight<ActualMastectomyweight*<Implantweight**

*Actual Mastectomy weight = calculated breast weight + 49.4

**Table 2:** Descriptive statistics for pre-operative breast weight, mastectomy weight, and implant weight

	Mean
Calculated breast weight Mastectomy weight Implant weight	291.2 ± 127.7 332.7 ± 178.0 373.8 ± 106.3
**Mean differences** Mastectomy weight and calculated breast weight Implant weight and calculated breast weight	49.4 ± 134.0 41.0 ± 130.2

**Implant weight = actual mastectomy weight + 41.0g

A linear regression was calculated to predict mastectomy weight and implant weight based on calculated breast weight. The regression equations are as follows:

$$\begin{array}{c} Predictedmastectomyweight=63.18+0.95\left(calculatedbreastweight\right)\\ Predictedimplantweight=209.66+0.56\left(calculatedbreastweight\right) \end{array}$$

**Figure 3:**
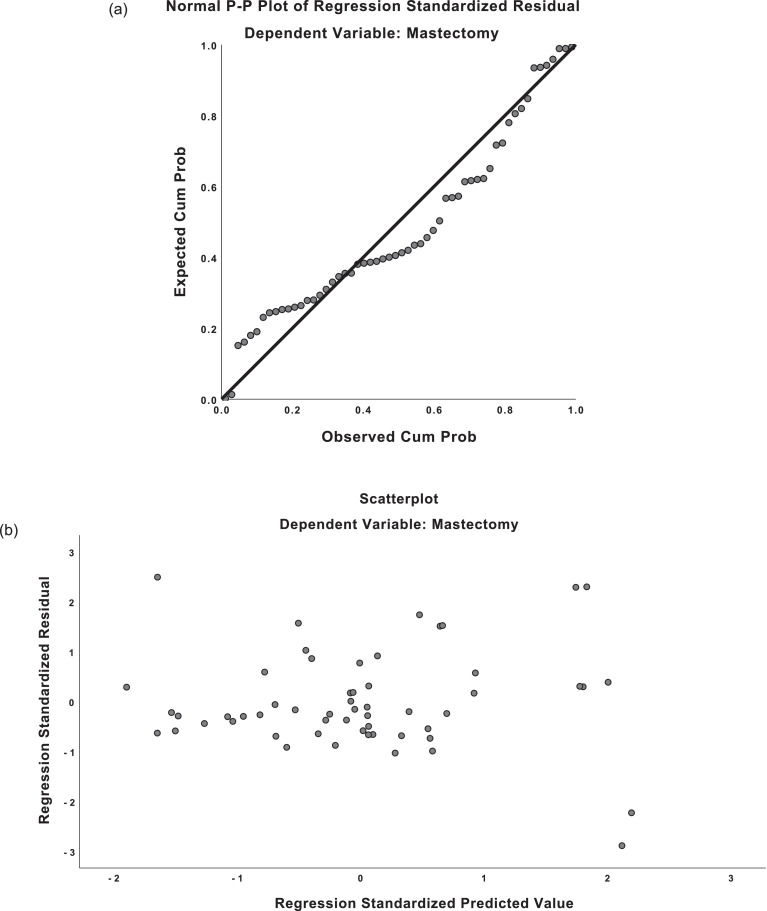

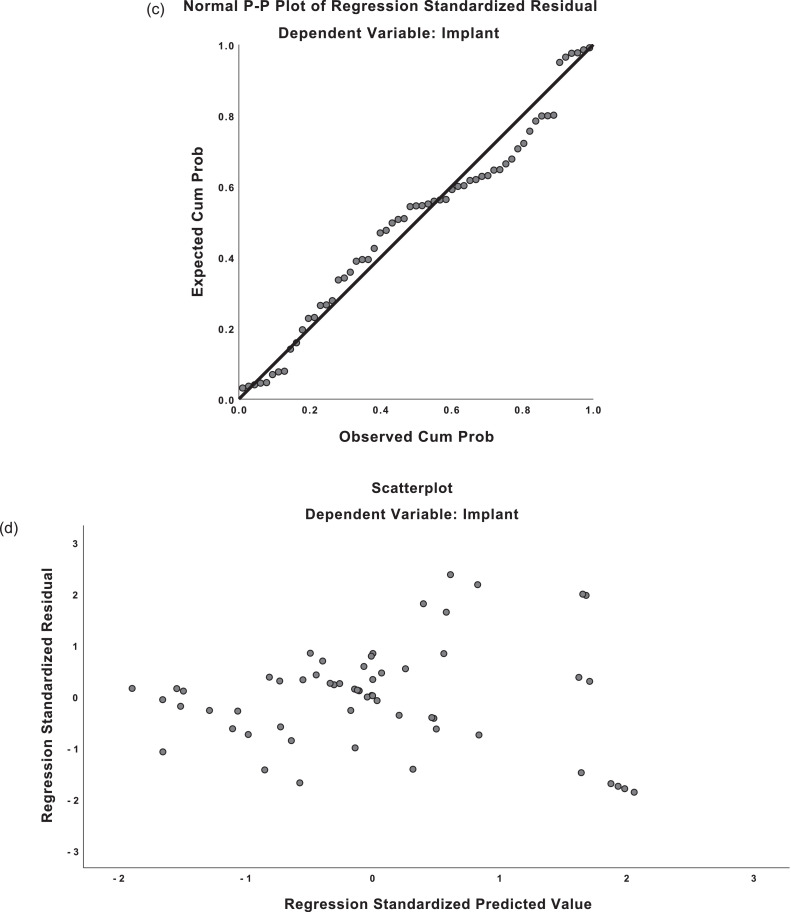
Predicted-probability (P-P) plots and residual plots for linear regression. **a)** P-P plot, mastectomy weight **b)** Residual plot, mastectomy weight **c)** P-P plot, implant weight **d)** Residual plot, implant weight

The regression equations demonstrated moderately strong correlation for both predicted mastectomy weight and implant weight (*R* = 0.659, *R* = 0.677). For mastectomy weight predictions, the regression equation was found (F(1,54)=41.551, p <0.001), with an R^2^ of 0.435. For implant weight predictions, the regression equation was found (F(1,57)=48.345, p <0.001), with an R^2^ of 0.459. The residuals from the regression were distributed normally on predicted-probability (P-P) plots. Scatterplots of the predicted values and residuals demonstrated homoscedasticity (see Figure 3). There was absence of multicollinearity with variance inflation factor (VIF) values of 1.000.

The mean predicted mastectomy weight was 332.7g (SD=117.4). The mean residual was 0g (SD=133.8). The mean predicted implant weight was 373.8g (SD=72.0). The mean residual was 0g (SD=78.2). There were 51/59 (86%) of standardized residuals for the mastectomy data within –1 to +1 on the residual plot; likewise, 44/59 (74%) of implant residuals were within –1 to +1 on the residual plot. This indicates that 86% of predictions using the mastectomy equation were within 133g of the actual mastectomy weight and 74% of implant size predictions were within 78g of the actual implant weight.

### Post-hoc Analysis

A post-hoc analysis was performed, where all cases where there was a 200g difference between implant weight and calculated breast weight were removed. The 200g difference was felt to represent one bra-cup-size difference^12^ and could suggest that these patients had expressed personal desire to be reconstructive larger or smaller than pre-operative breast size. Notably, 8/11 (73%) of these removed cases were noted to have either prior lumpectomy defects, breast deflation, or breast reduction surgery in preparation for NSM. Comparatively, 1/48 (2%) of the remaining cases had any of the aforementioned findings.

**Figure 4:**
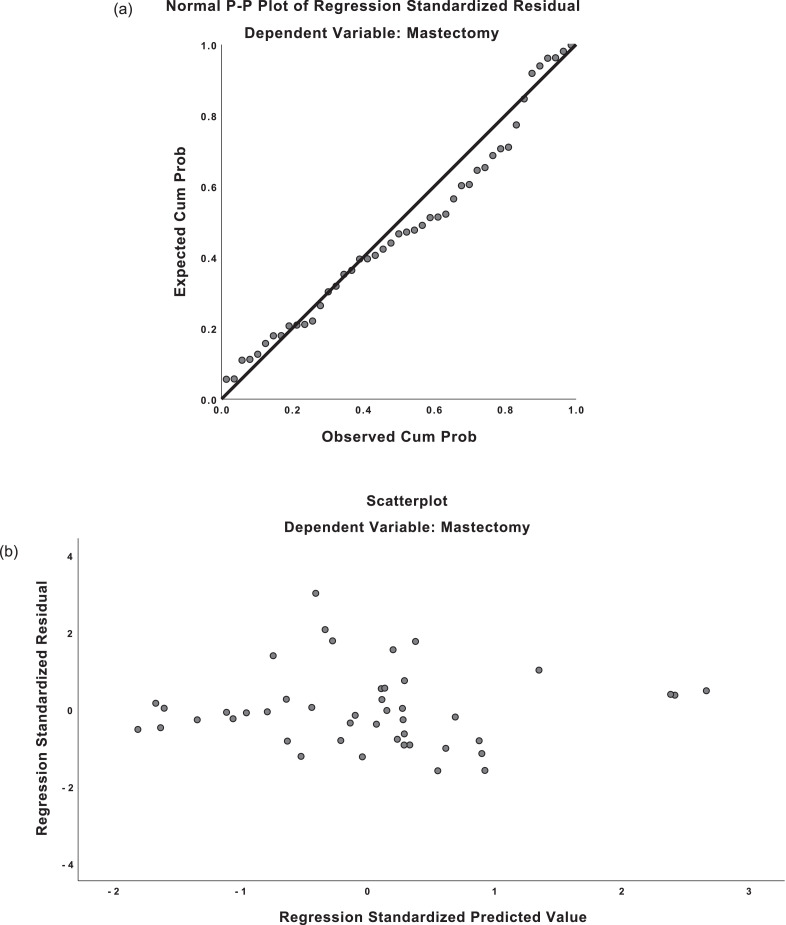

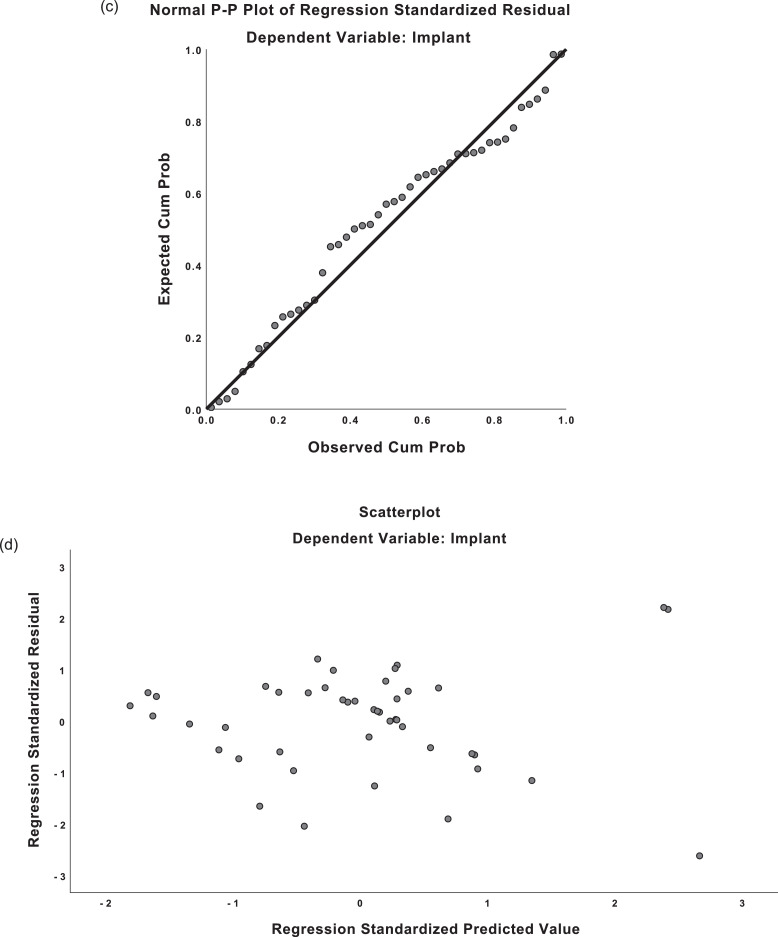
Post-hoc analysis: Predicted-probability (P-P) plots and residual plots for linear regression. **a)** P-P plot, mastectomy weight **b)** Residual plot, mastectomy weight **c)** P-P plot, implant weight **d)** Residual plot, implant weight

This post-hoc analysis resulted in a stronger correction for mastectomy weights (*R* = 0813) and for implant weights (*R* = 0.747). For mastectomy weight predictions, the regression equation was found (F(1,43)=83.593, p <0.001), with an *R^2^* of 0.660. For implant weight predictions, the regression equation was found (F(1,43)=54.353, p <0.001), with an *R^2^* of 0.558 (see Figure 4 for P-P plots and residual plots). As such, perhaps, a better regression equation for both mastectomy weight and implant weight *for women who prefer to be reconstructed to be similar size* would be as follows:

$$\begin{array}{c} Predictedmastectomyweight=8.43+1.08\left(calculatedbreastweight\right)\\ Predictedimplantweight=178.07+0.67\left(calculatedbreastweight\right) \end{array}$$

## DISCUSSION

Our study demonstrates how 3D imaging technology can be used to determine aesthetic breast size and make predictions for oncologic breast weight and implant size. It also establishes a relationship whereby the aesthetic breast size was less than the oncologic breast weight which was less than the implant size required to reconstruct the breast. From the calculated breast weight (as determined by pre-operative 3D imaging), we could apply the regression equations to estimate the mastectomy weight and implant size. Mastectomy weights fell within 133g of the value from the equation approximately 86% of the time. Implants fell within 78g of the value predicted by the equation approximately 74% of the time.

In our post-hoc analysis of our data, we attempted to improve the fit of our regression formulas to our data. We wondered if patient's preferences to be reconstructed larger or smaller than their pre-operative breast size contributed to diversity of the sample. Given that, we did not collect a priori data about patient size preference, we used a surrogate value of a 200g difference to suggest a one-cup-size difference in breast size – however, it is well acknowledged that the bra-manufacturing industry is not standardized and there is no valid volume that corresponds to one-cup-size.^12^ When cases with at least 200g difference between pre-operative breast volume and final implant size were removed, the correlation for our regression equations improved.

There was a difference in the percentage of patients with prior lumpectomy defects, deflation and pre-NSM breast reduction surgery among the cases removed and the cases that remained in the post-hoc analysis. The clinical relevance of prior lumpectomy defects is that the reconstructed breast may need to be sized differently to better match the patient's contralateral breast. Deflated breasts may need to be reconstructed larger than pre-operative size to better fill the stretched skin envelope.^13^ Patients who underwent staged breast reduction surgery prior to NSM had pre-operative breast size in excess of what is usually required for NSM and thus, would have been reconstructed smaller than pre-operative size.^14^ These findings suggest that our original data may have been heterogeneous with regards to patient characteristics and goals for desired breast reconstruction size. This is reflective of our overall practice to work with patients to meet their reconstructive goals, whether they wish to be reconstructed larger or smaller than pre-operative size.

Importantly, understanding patient's preferences for size of reconstruction is just one of multiple modifiable and non-modifiable factors that come into play when considering implant selection in the setting of mastectomy reconstruction. These include the patient's body mass index,^15^ native breast shape, amount of axillary tail of Spence tissue,^16^ oncologic surgeon's preference for mastectomy borders,^17^ potential ability to improve mild ptosis or deflation with a larger implant,^13^ and implant shape and gel characteristics. These factors should be examined in a larger study with the addition of prospective data.

Application of these equations to patient data in a pre-operative setting may help surgeons better prepare for surgery. Improved preparation with implant-based surgery could lead to shorter operative time, decreased use of sizers, improved infection rate, fewer mitigations (such as reinforcing borders), and a decreased learning curve for the novice surgeon.^18^

We were limited by our sample size. This was also a single center and single reconstructive surgeon study. As well, mastectomy borders may be different between breast surgeons and there were six different breast surgeons whose data were included. Breast ptosis varied from grade 1 to 2; it is a known limitation of Vectra 3D imaging that accuracy is decreased in more ptotic breasts due to the patient's standing position and positional masking of certain anatomic points.^19^ Post-operative Vectra 3D imaging was not performed – a comparison between post-operative calculated breast weight (using Vectra 3D imaging) and final implant size would help further our understanding of the relationship between calculated and reconstructed implant weight. Post-operative measurements would also allow us to investigate other parameters of breast shape, such as nipple position changes with surgery. Another limitation is that mastectomy specimens may differ in terms of amount of glandular verusus fatty tissue and differences in composition could affect specimen weight. In our formula, failure to account for these composition differences could have contributed to the residuals that were found. However, given that, it is difficult to accurately assess breast composition pre-operatively, we did not feel that differences in breast composition could be included in our predictive formula.

In our post-hoc analysis, our model attempted to include consideration for patient surgical goals, such as the desire to be reconstructed larger or smaller than pre-operative size using a weight-based surrogate. However, this would have been better controlled if we had excluded any patient who did not wish to be reconstructed in a similar breast size in an a priori manner.

## CONCLUSIONS

Our study demonstrates that even in an ethnically diverse population with a broad range of breast size, 3D imaging technologies can be used as an additional tool to predict final implant sizes. A better understanding of the relationship between the 3D predicted aesthetic breast size and the final implant selection provides additional objective guidance to for the surgeon to improve their pre-operative planning. The regression technique used here may be utilized again in the future with consideration of additional variables to further refine the regression equations and improve predictability. This would represent an avenue for quality improvement. Other surgeons may wish to apply this same technique to create regression formulas for their own data to create a personalized equation for their own set of patient data.

With the considerations outlined in this study, 3D imaging technologies may be a useful tool to predict implant sizes for direct-to-implant breast reconstruction.
